# The Stage of the Estrus Cycle Is Critical for Interpretation of Female Mouse Social Interaction Behavior

**DOI:** 10.3389/fnbeh.2020.00113

**Published:** 2020-06-30

**Authors:** Trishala Chari, Sophie Griswold, Nick A. Andrews, Michela Fagiolini

**Affiliations:** Neurodevelopmental Behavior Core, Boston Children’s Hospital, Boston, MA, United States

**Keywords:** social behavior, estrus cycle, learning and memory, anxiety, sex differences

## Abstract

Female animals in biomedical research have traditionally been excluded from research studies due to the perceived added complexity caused by the estrus cycle. However, given the importance of sex differences in a variety of neurological disorders, testing female mice is critical to identifying sex-linked effects in diseases. To determine the susceptibility of simple behaviors to hormonal fluctuations in the estrus cycle, we studied the effects of sex and the estrus cycle on a variety of behavioral tasks commonly used in mouse phenotyping laboratories. Male and female C57BL/6J mice were tested in a small battery of short duration tests and, immediately on completion of each test, females were classified using cytology of vaginal lavages as sexually-receptive (proestrus and estrus) or non-receptive (NR; metestrus and diestrus). We showed that there was a significant difference in 3-chamber social interaction (SI) between female mice at different stages of their estrus cycle, with sexually-receptive mice showing no preferential interest in a novel female mouse compared with an empty chamber. NR female mice showed the same level of preference for a novel female mouse as male mice did for a novel male mouse. No differences between or within sexes were found for tests of anxiety elevated plus maze (EPM; Hole board), working memory [Novel object recognition (NOR)], and motor learning (repeated tests on rotarod). We conclude that the stage of the estrus cycle may impact SI between same-sex conspecifics, and does not impact performance in the elevated plus-maze, hole board, NOR, and rotarod.

## Introduction

In 1993, NIH scientists resolved to require the inclusion of women in clinical trials through the NIH Revitalization Act (NRA), positing that exclusion of women from clinical research is problematic due to the broad implications of biological sex differences. NRA has proven a success in this domain—in the years since its inception, clinical research participants have nearly achieved gender parity. Unfortunately, sex bias persists in animal research, as a National Institutes of Health (2015) *Guide* notice asserted (NOT-OD-15-102). Female animals are still commonly excluded on the presupposition that they are intrinsically more variable than males due to the fluctuation of hormones across their estrus cycle which typically lasts 4 days in mice (Beery and Zucker, 2011). Although the question of the estrus cycle’s effect on behavior has not been fully addressed, researchers still commonly cite the potential variability it may introduce as a primary reason for excluding female animals (Prendergast et al., 2014).

The exclusion of female animals in research has broad ramifications across biomedical and neuroscience research. Despite known sex differences in the prevalence and presentation of several neuropsychiatric disorders including depression and anxiety disorders, male animals are often used in neuroscience and behavior research to the exclusion of females (Beery and Zucker, 2011). Further, many articles across fields fail to report the sex of non-human research subjects altogether (Kilkenny et al., 2010; Beery and Zucker, 2011). The prevalence of this sex bias belies an all too common assumption that data derived from males can be directly translated to females. Given commonly observed sexual dimorphisms across several neuropsychiatric and behavioral domains, researchers that include animals of only one sex may erroneously generalize sex-linked effects and fail to observe others.

Accounting for behavioral differences attributable to the estrus cycle remains an important step in encouraging researchers to use animals of both sexes. Comprehensive literature cataloging such behavioral sexual dimorphisms would represent a powerful tool for researchers otherwise inclined away from using females in their work. While the literature on the impact of the estrus cycle on the behavior of commonly used lab animals remains relatively sparse, many recent studies have sought to address this enduring problem.

A review of 293 journal articles assessing behavioral, morphological, physiological, and molecular traits in male and female mice found that females were not significantly more variable than males by any of the metrics assessed (Prendergast et al., 2014). Additionally, a review of 311 journal articles by Becker et al. (2016) found that variability in brain function across several domains was not significantly different between male and female rats. While these reviews have not pointed to significant variability in behavior and brain function between males and females, a behavioral study by Meziane et al. (2007) found a strain-dependent effect of behavioral variability during different stages of the estrus cycle. While BALB/cByJ female mice demonstrated significant behavioral variability depending on the stage of the estrus cycle in the open field, tail-flick, and tail suspension tests, female C57BL/6J (C57) mice displayed stable behavior across the estrus cycle in the open field and tail-flick tests but the tail suspension test showed distinct variability (Meziane et al., 2007).

**Figure 1:**
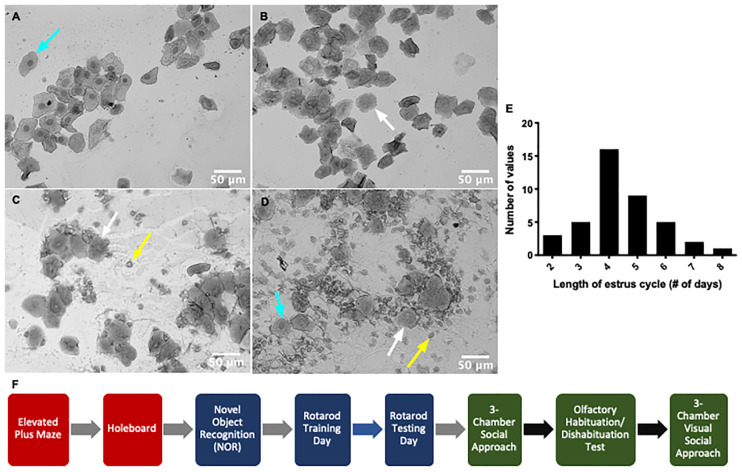
Timeline of the mouse estrus cycle. The mouse estrus cycle occurs over 2–8 days as indicated by the histogram representing the length of estrus cycle for *n* = 41 female mice **(E)** and is represented by four distinct stages: proestrus **(A)**, estrus **(B)**, diestrus **(C)** and metestrus **(D)**. Each stage of the cycle is distinguished using vaginal cytology and identification of specific cell types (leukocytes—yellow arrow, nucleated epithelial cells—cyan arrow, cornified epithelial cells—white arrow). Mice in proestrus and estrus are sexually receptive (SR) and mice in diestrus and metestrus are non-receptive (NR). **(F)** The timeline of behavioral testing shows the order of behavioral tasks performed. Anxiety behavioral tasks (red) were first carried out to prevent any effect of handling on the behavioral output. This was followed by learning and memory tests (blue) and then sociability-related tests (green). Gray arrows indicate the subsequent behavioral task was carried out atleast 24 h to a week later. Black arrows signify the next task was carried out at least a week to 2 weeks later. Blue arrow denotes that rotarod testing was carried out 24 h after rotarod training.

These articles provide insight into the potential impacts of the estrus cycle on behavioral and neurological variability in female animals, yet literature in which researchers have tracked the estrus cycle throughout behavioral testing remains sparse at best. To address this issue of behavioral variability across the estrus cycle, we have conducted a battery of behavioral tests using freely cycling C57 female mice while tracking their estrus cycle *via* vaginal lavage and cytological evaluation (Figures 1A–E). Here, we report the effects of sex (male vs. female mice) and the estrus cycle on a variety of behavioral tasks [3-chamber social interaction (SI), elevated plus maze (EPM), hole board, Novel object recognition (NOR) and rotarod] commonly used in mouse phenotyping laboratories to determine the susceptibility of these behaviors to hormonal fluctuations.

## Methods

### Animals

C57BL/6J mice [The Jackson Laboratory (Bar Harbor, ME, USA)] were housed in same-sex groups of 3–5 with food and water available *ad libitum*. Mice were housed on a 12-h light/dark cycle (lights on from 07:00 h to 19:00 h) at 23°C with controlled humidity (45–55%). All experiments were approved by the institutional animal care and use committee (IACUC) at Boston Children’s Hospital and performed in compliance with all NIH guidelines for the humane treatment of animals. Mice were tested in all behavioral paradigms between postnatal day 60 and 90 (P60–90). Male and female mice were tested on separate days to account for sensitivity to pheromones during behavioral testing.

### Mouse Estrus Cycle Stage Identification

Estrus cycle monitoring and stage classification was done using vaginal cytology described previously (McLean et al., 2012). Stages of the estrus cycle were determined based on observation of leukocytes, cornified epithelial cells, and nucleated epithelial cells (Figures 1A–D, Byers et al., 2012). Mice were lavaged daily for 2 weeks before testing to ensure the timeline of the estrus cycle was normal for each mouse. The estrus cycle lasted 2–8 days with 4 days being the most frequent cycle length (Figure 1E). A lavage from each animal was then taken immediately after each behavioral test. Lavage administration after testing minimizes the risk of additional stress, which can influence the behavioral performance of the animal (Ekambaram et al., 2017). Mice were assigned an estrus cycle stage based on cytological evaluation and then delegated to an experimental group [sexually-receptive (estrus and metestrus) and non-receptive (NR; diestrus and proestrus)] for each behavioral test (McLean et al., 2012).

### Behavioral Testing

All of the tests chosen for study (except for the rotarod test which was performed over 2 days) were ones that did not take multiple days to complete since the estrus cycle stage may change from 1 day to the next. A total of 41 female mice and 18 male mice were subject to a battery of tests performed in a particular order (Figure 1F). Some behavioral tests did not require the use of all 41 female mice since the results for those tests showed minimal variability across cohorts tested. As such, not all females went through all behavioral tests. All mice followed the same order of testing, but some skipped certain behaviors or ended on an earlier behavioral test. Because the stage of the estrus cycle was determined immediately following each test, all testing was performed blinded. The testing order was not randomized for sex to prevent cross-contamination of pheromones. However, the testing was randomized for the stage of the estrus cycle since not all mice were in the same stage at any one time. This also accounted for the different numbers of females used for each test since females could be disproportionately allocated to one group over another. Therefore, some tests required an additional number of female mice to balance the two female experimental groups. Anxiety-related behavioral tasks were tested first to avoid the effect of handling on the behavioral output of the mice (Figure 1F).

### Anxiety

**Figure 2:**
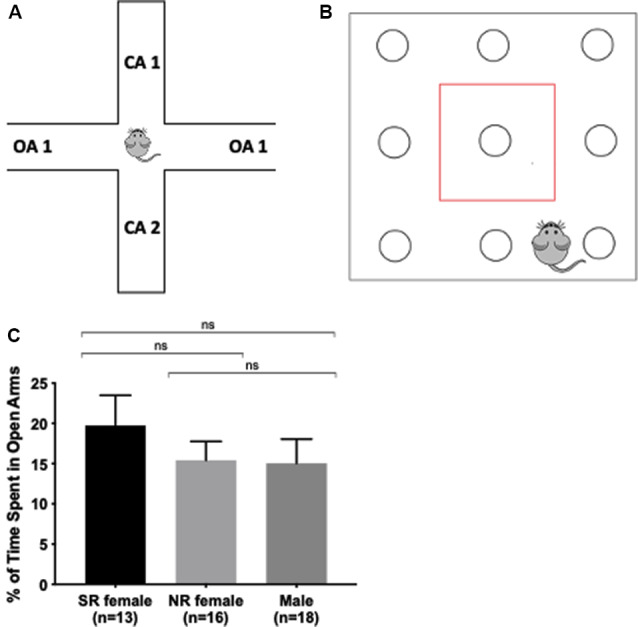
Anxiety is not influenced by the estrus cycle. Apparatus for the elevated plus-maze. Mouse spends 5 min exploring two open arms without walls and two closed arms enclosed by walls. The more time spent in the open arms, the less anxiety and stress the mouse exhibits **(A)**. Apparatus for hole board. Mouse spends 15 min exploring a square arena containing exploratory holes for nose pokes. The more total time in, distance traveled in, and the number of entries into the center (enclosed in the red box), the less anxious the mouse. The more basic movements, rearing, and the number of pokes indicate greater exploratory behavior **(B)**. There was no significant difference between the female and male groups in the percent (%) of time spent in the open arms **(C)**. ns > 0.05.

Elevated Plus Maze (EPM) and hole board testing were used to measure anxiety in mice. These tests examine the exploratory behavior of mice in unfamiliar surroundings mimicking what is considered anxious behavior in humans (Lezak et al., 2017). EPM entails placing the mouse in an arena with two enclosed and two open arms (35 cm long) elevated 50 cm above the floor for 5 min. At the end of each trial, the maze was cleaned with Clidox to eliminate the previous test mouse odor. Exploratory behavior was recorded and measured using Ethovision XT, v11.5 software (Noldus, Netherlands). The percent of time spent in the open arms is an indicator of anxiety (Figure 2A).

The hole board test consisted of a square arena (45 × 45 cm) with nine holes in the floor (Figure 2B). Two layers of infrared beams formed a grid across the arena to measure horizontal and vertical activity and further infrared beams located in the holes in the floor records when a mouse pokes its nose into the hole. Testing lasted 15 min with various parameters recorded to assess exploratory behavior (Supplementary Table S1). The arena was cleaned with Clidox between each mouse.

### Learning and Memory

**Figure 3:**
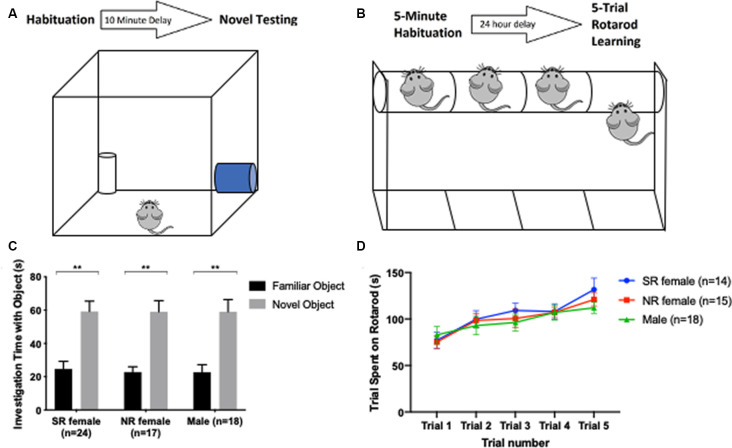
Short-term memory and motor learning are not influenced by the estrus cycle. Timeline and apparatus for the novel object. Mouse initially habituates to two identical objects (trial ends after a total of 20 s spent interacting with both objects). After 10 min, the mouse is placed back into the novel object arena for 5 min with a novel object and one of the two objects it habituated to **(A)**. Timeline and apparatus for rotarod motor learning task. Mouse initially habituates walking on a rotarod beam for 5 min. After 24 h, the mouse undergoes five consecutive trials, and time spent on the platform before falling off is recorded per trial **(B)**. Both female groups and males showed a significant preference for a novel object over a familiar object **(C)**. In the rotarod task, all groups spent more time on the rotarod from one trial to the next, as expected for C57 mice, and did not show significant difference among one another for time spent on the rotarod in each trial **(D)**. **<0.005.

Novel Object Recognition (NOR) is regarded as a short-term test for measuring non-hippocampal dependent working memory (Cohen and Stackman, 2015). Mice were exposed to two identical white plastic cylinders in a 40 × 40 cm arena until they had explored both objects for a total time of 20 s. After a 10-min delay, mice were returned to the arena where one of the objects had been replaced with a blue glass cylinder. Interaction times with the familiar and novel objects were recorded (Figure 3A). Mice were excluded from the testing with the novel object if they did not reach the criterion of 20-s exploration time with the identical objects within a 5-min duration (Leger et al., 2013). All behavior was recorded using Ethovision XT, v.11.5. The arena was cleaned with Clidox between each mouse.

Motor learning was measured using the rotarod test (Shiotsuki et al., 2010). On day 1, mice were trained to run on a rotating beam [four rotations per minute (rpm)] for 5 min. After a 24-h delay, mice were placed on the moving beam as it accelerated by 1 rpm per second from a starting speed of 4 rpm. Testing was repeated five times (inter-trial interval of 3 min) and latency to fall off of the beam was recorded for each of the five trials and the average of the five trials taken as the performance value (Figure 3B). Equipment was cleaned with Clidox between each mouse tested.

### Sociability

**Figure 4:**
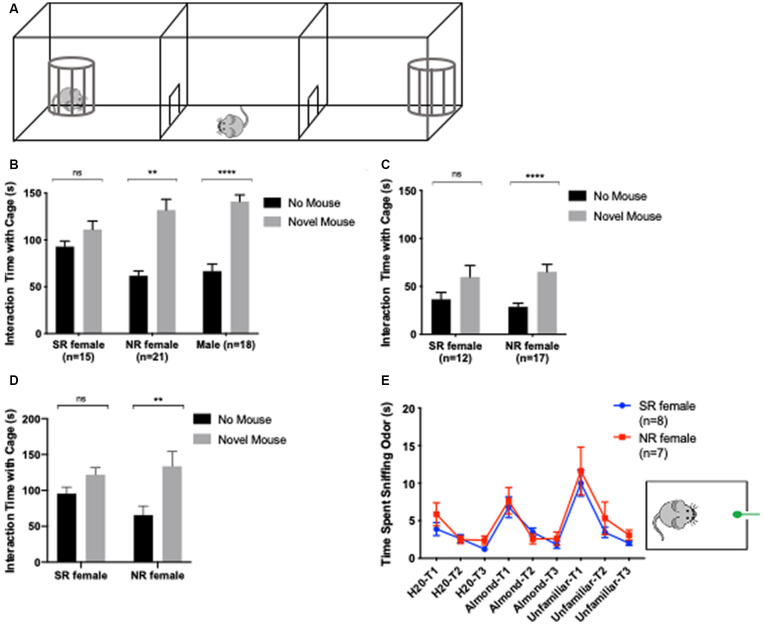
An estrus cycle effect on the social behavior of female C57BL/6J mice. Apparatus for 3-chamber social interaction (SI). Cages for SI are traditionally barred **(A)**. In the SI task, NR females and males show a significant preference for novel same-sex mouse over a cage without a mouse. Sexually-receptive (SR) females show no preference for a novel mouse cage or a cage without a mouse **(B)**. In a visual SI task, where the cages are enclosed not barred, SR females still demonstrate an absence of preference for the novel mouse over a cage without a mouse **(C)**. Ten female mice were retested a second time in SI to compare interaction time when NR vs. SR. Two-way repeated-measures ANOVA was used with *post hoc* Bonferroni’s test to compare interaction time with novel vs. no mouse for each group **(D)**. Olfaction habituation/dishabituation test was used to determine differences in odor detection between NR and SR females. A mouse is exposed to cotton buds with odor in a habituated cage. SR and NR females demonstrate no significant difference in odor detection for distilled water (H_2_O), almond extract, and odor from a novel, unfamiliar same-sex mouse **(E)**. **<0.005, ****<0.0001, ns > 0.05.

Sociability was measured using the 3-chamber social approach task (Figure 4A). The experimental protocol was adapted from Yang et al. (2011). Mice habituated for 5 min to a 60 × 40 cm arena divided into three chambers by two panels. The left and right chambers each contained an empty cage in one corner. Mice were then returned to the middle chamber and blocked from the left and right chamber while a novel mouse was randomly placed in the left or right chamber cage. The novel mouse was of the same sex, age (P60–90), and strain and was not a sibling of the test mouse. After the novel mouse was introduced, the test mouse was allowed to explore all three chambers for 10 min. The arena was cleaned with Clidox in between testing each mouse to eliminate urine and any odor from the previous test and novel mouse. To study the effect of removing odor and tactile cues on SI, a visual SI protocol was designed that differed slightly from the 3-chamber social approach. This was done by placing the novel mouse in an enclosed cage rather than a cage with bars as used for the initial 3-chamber task. A repeated test for the 3-chamber social approach was performed at least 2 weeks after the first 3-chamber social approach or at least 1 week after the visual social approach was completed (Figure 1F). Behaviors were recorded *via* a video camera and tracked using Ethovision XT, v11.5 software (Noldus, Netherlands).

Odor discrimination was tested with the olfactory habituation/dishabituation test (Figure 4E; Yang and Crawley, 2009). The mouse is placed in an empty home cage with clean wood bedding and allowed to acclimate for 30 min. The mouse is then presented with an odor on a cotton bud for 2 min followed by a 1-min inter-trial interval. Each odor type is present three times consecutively before presenting the next type of odor. The odor types were presented in the following order: distilled water (H_2_O), 1:100 dilution of almond extract, and an unfamiliar, same-sex mouse. The odor from an unfamiliar mouse was acquired by swiping the unfamiliar mouse’s cage bedding with a cotton bud. Behaviors were recorded *via* a video camera and a stopwatch was used to track the amount of time the mouse spent sniffing the odor.

### Statistics

Statistical analyses were performed using GraphPad Prism (Prism 7). Data are shown as mean ± standard error of the mean (SEM). To examine significant differences between means, two-way analysis of variance (ANOVA) with repeated measures (RMs) was used for repeated SI. One-way ANOVA was used for EPM and hole board and one-way with RMs for rotarod. Paired student *t*-tests were used for the 3-chamber social approach and NOR. *Post hoc* tests included Bonferroni’s in repeated SI. Tukey’s multiple comparisons test was used after all one-way ANOVAs.

## Results

Given that certain behavioral phenomena might differ between sexes and across the estrus cycle in females, we sought to investigate the contributions of the estrus cycle stage to behavioral changes in mice. We tested female mice across the estrus cycle and male mice in a variety of behavioral tasks that measure anxiety, learning, and memory and sociability.

### Estrus Cycle in Rodents

We characterized the estrus cycle stage of female mice by acquiring vaginal samples immediately after each behavioral task and examining their cytology under a microscope. Each stage was characterized based on the proportion of leukocytes, cornified epithelial cells, and nucleated epithelial cells (Figures 1A–D). The proestrus stage of the estrus cycle is dominated by nucleated epithelial cells whereas cornified epithelial cells are largely abundant in the estrus stage. The metestrus stage shows a mixture of all three cell types but mainly consists of cornified epithelial cells. The diestrus stage also shows a mixture of all cell types with leukocytes as the largest proportion. The progression of the cycle suggests leukocytes aggregate during diestrus and disappear with the onset of proestrus. During proestrus, nucleated epithelial cells appear and are replaced by cornified epithelial cells when mice enter the estrus stage. By metestrus, leukocyte invasion begins and continues into diestrus (McLean et al., 2012). Mice found to be in proestrus and estrus were placed into one grouping and mice in diestrus and metestrus were combined into another grouping for purposes of analysis since the former grouping is considered receptive to males and the latter are not.

### Anxiety

We first tested anxiety-related sex differences as the effect of the estrus cycle on anxiety remains inconclusive in the literature. Furthermore, anxiety-related behavioral tasks are sensitive to handling and should, therefore, occur before other tests. Male and female mice were tested on EPM as an assay for anxiety (Rodgers and Johnson, 1995; Lezak et al., 2017). There was no significant difference in percent time spent in the open arms between sexually-receptive females, NR females, and males (*F*_(2,44)_ = 0.5130, *p* = 0.4888; Figure 2C). Additionally, we tested mice on the hole board, a behavioral task measuring multiple behaviors including anxiety and exploratory activity. There was no significant difference in the hole board among all parameters (*p* > 0.05) except for the number of pokes in the center zone. There was a significant difference in the number of pokes into the center of the arena (*F*_(2,52)_ = 4.771, *p* = 0.0125) and *post hoc* tests showed that the number of pokes into the center zone was different between males and sexually-receptive females and between males and NR females (Supplementary Table S1). Overall, the results show no differences in anxiety within females during different estrus cycle stages and across sex.

### Learning and Memory

Given that estrogen may modulate short-term memory and learning *via* activation of estrogen receptors, we sought to investigate if females show differences in learning and memory across their estrus cycle (Liu et al., 2008; Frick, 2009; Han et al., 2013; Bean et al., 2015). Mice were tested in the NOR task for short-term non-hippocampal memory and in the rotarod task for motor learning. There was no difference between males and females in NOR (*F*_(2,106)_ = 0.2567, *p* = 0.7742). A significant difference between time spent with a novel object and time spent with a familiar object was found for each group: sexually-receptive females (*t*_(17)_ = 3.3, *p* < 0.005), NR females (*t*_(13)_ = 5.74, *p* < 0.005) and males (*t*_(17)_ = 3.72, *p* < 0.005; Figure 3C).

With respect to motor learning, there was a significant difference across trials for sexually-receptive females (*F*_(2.827,36.75)_ = 6.934, *p* = 0.001), NR females (*F*_(3.449,48.29)_ = 6.959, *p* = 0.0003) and for males (*F*_(2.784,47.32)_ = 5.806, *p* = 0.0023; Figure 3D).

### Social Interaction

Finally, we tested mice for sex differences in SI. The estrus cycle has been well- established in modulating sexual behavior and can affect how a mouse approaches a novel mouse of the same vs. different sex (Kim et al., 2016). We used the 3-chamber social approach task to determine the sociability of our test mice with a novel, same-sex mouse. We found that NR females performed similarly to males in this task showing a greater interaction time with a novel mouse compared with an empty cage (NR females: *t*_(20)_ = 5.123, *p* < 0.0001, males: *t*_(17)_ = 6.575, *p* < 0.0001). In contrast, sexually-receptive females showed no difference in interaction time between the novel mouse and an empty cage (*t*_(14)_ = 1.475, *p* = 0.1624; Figure 4B). Having found this difference, we first tested whether the difference in preference for the novel mouse was due to differences in olfactory perception between sexually-receptive and NR females. This was done with the olfactory habituation/discrimination task where we found that both sexually-receptive and NR females responded equally to the presentation of different odors (*p* > 0.05 per each odor; Figure 4E). We then tested the effect of removing odor and tactile cues by placing the novel mouse under a cage made from clear, solid acrylic (compared with the standard cage of acrylic bars). The interaction time with a novel mouse compared to an empty cage was not significantly different (*t*_(11)_ = 2.028, *p* = 0.0675) in sexually-receptive females when only the visual cue of a novel mouse was presented and both olfactory and tactile cues of a novel mouse were eliminated. NR females continued to show a significant preference for novel mouse over an empty cage in the absence of tactile and odor cues (*t*_(16)_ = 5.364, *p* < 0.0001; Figure 4C). These data suggest that the difference in SI between sexually-receptive and NR females is not dependent on odor or tactile cues from the novel mouse but rather visual cues. Finally, we confirmed that this difference in SI performance is present in individual mice when they switch between stages. Mice were tested twice, specifically during a NR and a sexually-receptive stage and each time with a distinct novel mouse. Here, there is a significant preference for the novel mouse over a cage without a mouse when females were NR and when they were sexually-receptive (*F*_(1,18)_ = 9.731, *p* = 0.0059). However, *post hoc* testing indicated a significant effect of receptivity, such that NR females interacted more so with a novel mouse than when they were sexually receptive (SR; *p* = 0.0026; Figure 4D). These results lead to the conclusion that there is a difference in female social preference for a novel, same-sex mouse across the estrus cycle. Furthermore, this difference may be linked to the visual cues of a novel mouse and appears independent of odor and tactile cues.

## Discussion

Our primary finding is that sexually-receptive female mice do not show a statistically significant preference for a novel mouse compared to an empty enclosure. This is in direct contrast with NR females and males that instead spend more time exploring the novel mouse. These results were not due to differences in olfactory perception, because both receptive and NR mice performed similarly in the olfactory discrimination/habituation task (Figure 4E). There were no differences between any of the experimental groups regarding behavioral performance in any of the other tests we analyzed suggesting no differences in anxiety, short-term non-hippocampal working memory, and motor learning.

Anxiety is often comorbid with and related to sex differences in humans. Women have twice as high an incidence of anxiety-like symptoms than men (Nolen-Hoeksema, 2001; Bekker and van Mens-Verhulst, 2007). The reproducibility of this bias in rodents remains contentious, primarily because anxiety-like symptoms differentiated between sex cannot be reproduced in animal models (Lezak et al., 2017). There are reports describing experiments that have evaluated sex bias of rodent models in the context of anxiety-like behavior. However, the conflicting conclusions on sex bias in these experiments have made it difficult to surmise whether rodents demonstrate a sex difference concerning these behaviors (Donner and Lowry, 2013; Mehta et al., 2013; Kokras et al., 2015). Sensitivity to drugs may also be impacted by the cycle. Indeed, Picard et al., 2019 recently showed that C57Bl6/J females in estrus do not respond to low-dose ketamine, while NR females do and demonstrate a response comparable to males.

Our results differ from previous anxiety studies that may have been susceptible to the influence of the lavages on female rodents’ stress (Gouveia et al., 2004; D’Souza and Sadananda, 2017). Similar to our findings, Meziane et al. (2007) and Bath et al. (2012) found no difference among estrus cycle stages of C57 mice in EPM and open field behavioral performance when lavages were taken within 10 min after testing ended. These results coincide with the results presented here and suggest female rodents do not show specific anxiety differences across the estrus cycle and in comparison with males. Our study of the hole board did find significant differences between the sexes concerning pokes in the center zone. However, the remaining parameters measured for the hole board did not change significantly suggesting that sexual receptivity does not have a profound effect on the exploratory drive.

There was no discernible effect of the estrus cycle on short-term working memory in mice. Since studies have found a critical window for short-term memory modulation in relation to estrogen receptor activation, the estrus cycle may not influence behavior at later stages of the rodent lifespan including P60–90 when we tested our mice (Bean et al., 2015). Possibly the levels of estrogen across the estrus cycle are not high enough to activate estrogen receptors in the forebrain. Previous studies that applied estrogen exogenously for treating cognitive decline performed these treatments in either ovariectomized or aged mice. The increased levels after exogenous treatment may exceed basal levels of estrogen across the typical estrus cycle stages but were not measured in any of the studies described (Liu et al., 2008; Frick, 2009; Han et al., 2013; Bean et al., 2015). Typically, estrogen levels in low-receptivity phases (diestrus and metestrus) are lower than high-receptivity phases (estrus and proestrus), but not eliminated (McLean et al., 2012). Therefore, it would be interesting to quantify estrogen receptor activation with basal levels of estrogen for each cycle stage in comparison to activation with estrogen treatment. Together, these are new approaches we can take to determine the extent of hormonal changes across the estrus cycle on short-term non-hippocampal memory.

The results from the rotarod study concur with previous studies of motor learning in which behavioral performance remained unaffected by different stages of the estrus cycle and by sex (Meziane et al., 2007). Based on these results, hormone fluctuations during the estrus cycle are not potent enough to affect motor learning. Exogenous administration of hormones may prime activation of learning in motor-based tasks, but healthy females do not demonstrate a difference in motor learning in comparison to males at different stages of the estrus cycle (Morgan and Pfaff, 2001; Ogawa et al., 2003). Since locomotion in the hole-board task was not different between sexually-receptive and NR females and males, the results from rotarod were dependent on motor learning and not locomotor activity (Supplementary Table S1).

Generally, studies of rodent social behavior have focused on the effect of the hormones vasopressin and oxytocin. However, it is important to consider differences in social behavior concerning hormonal fluctuations occurring throughout the estrus cycle because of its effects on sexual behavior. Mounting behavior of C57Bl6/J male mice on females is significantly higher when females are in estrus than diestrus, signaling that sexual receptivity coincides with mating behavior (Powers, 1970; Kim et al., 2016). A limited number of studies have shown the effect of estrus cycle on social behavior. One such study has focused on the aspect of social learning, in which a mouse shows a preference for food that was previously detected from a conspecific’s breath (Ervin et al., 2015). During the sexually-receptive, proestrus stage of the estrus cycle, a female mouse will show a preference for food that it recognizes from a conspecific more so than a mouse in NR diestrus (Choleris et al., 2011). For our experiments, we wanted to separate the estrus cycle effects on memory from sociability. However, for future experiments, it would be interesting to consider the effects on social memory, such as in the 3-chamber social recognition task (Yang et al., 2011). If sexually-receptive females are still able to distinguish between familiar and novel mice in this task but show reduced sociability then this would fall in line with our results on learning and memory and sociability.

We hypothesize that a SR mouse may be less likely to interact with a novel same-sex mouse given that the mouse is primed for reproduction and will actively be searching for a sexual partner (Kim et al., 2016). This is not the case for NR mice, perhaps because their motivation for social novelty outweighs their motivation for finding a mate. A recent study showed that increased estrogen levels can act on the somatosensory cortex to increase the excitability of parvalbumin (PV) fast-spiking interneurons (Clemens et al., 2019). This role of estrogen may potentially extend to other cortical areas, such as the primary visual cortex. We, therefore, also hypothesize that the estrus cycle may affect visual cortical activity during SI such that the hormone profile during sexual receptivity regulates PV neuron firing.

The sensitivity of social behavior to the estrus cycle may have implications in neurodevelopmental disorders and neuropsychiatric diseases. Traditionally, 3-chamber social approach has been used to identify deficits in sociability in mouse models of autism, including Fragile X Syndrome, Rett’s Syndrome, and 16p11.2 deletion (Moretti and Zoghbi, 2006; Dahlhaus, 2018; Stoppel et al., 2018). Female mice are generally excluded from social behavioral tasks, but testing females from these models may shed light on the sexual dimorphism of sociability in autism spectrum disorders (ASDs; Silverman et al., 2010). A deficit in SI between the sexes has also been demonstrated in the context of autism, but sexual receptivity may be a factor that differentially regulates sociability and further necessitates the monitoring of a mouse’s estrus cycle (Jeon et al., 2018). The estrus cycle should be considered moving forward with future experiments on autism mouse models.

Given the strain-dependent effects of the estrus cycle found by Meziane et al. (2007) in their study, we acknowledge that the use of a single strain of mouse (C57BL/6J) represents an important limitation on our findings. Further, the classification of mice as either SR in proestrus and estrus and NR in metestrus and diestrus, limits the granularity of our analysis, particularly surrounding our findings with regards to SI. Future work might address these limitations in examining the behavioral impacts of the individual hormones that fluctuate throughout the estrus cycle.

While female animals remain under-represented in biomedical research out of concern for variability introduced by the estrus cycle, the literature exploring the link between female hormone cycling and variability across many domains remains sparse. The studies described here using C57BL/6J mice suggest that hormone cycling may be responsible for differences in SI in the 3-chamber social approach test, but the underlying mechanism requires further study. We have only studied one common strain of mouse in these experiments and expanding the range of strains would be enlightening. An extensive literature on variability introduced by the estrus cycle across species and between strains would represent a powerful tool for researchers. Such literature would allow researchers to account for potential variability introduced by the estrus cycle and allow for a fuller realization of the NIH Revitalization Act.

## Data Availability Statement

The raw data supporting the conclusions of this article will be made available by the authors, without undue reservation.

## Ethics Statement

The animal study was reviewed and approved by Institutional Animal Care and Use Committee, Boston Children’s Hospital.

## Author Contributions

TC and SG conducted all behavioral experimental and vaginal cytology. NA helped with some of the behavioral experiments. TC analyzed most of the data and wrote the article. SG provided additional writing and analysis to the article. NA and MF provided additional writing to the article and revisions. TC, SG, NA, and MF contributed to writing. TC and SG performed experimental data collection. NA and MF conceived the initial idea.

## Conflict of Interest

The authors declare that the research was conducted in the absence of any commercial or financial relationships that could be construed as a potential conflict of interest.
