# Supplementary material for: The Stage of the Estrus Cycle Is Critical for Interpretation of Female Mouse Social Interaction Behavior
Source: Front Behav Neurosci. 2020 Jun 30;14:113. doi: 10.3389/fnbeh.2020.00113 (PMC7340104; doi:10.3389/fnbeh.2020.00113)
Supplement: Supplementary file 1 [file Table_1.pdf]

Holeboard measure	Mean ± Standard error of mean (SEM)	One-way ANOVA statistics
Basic movements (total # of beam breaks)	SR female: 3028 ± 185.1<br>NR female: 3037 ± 195.3<br>Male: 2827 ± 124.6	($F(2,52)=0.4741$, $P>0.05$) ns
Rearing (# of vertical beam breaks)	SR female: 66.11 ± 7.75<br>NR female: 88.11 ± 13.37<br>Male: 65.60 ± 6.14	($F(2,52)=1.887$, $P>0.05$) ns
Distance in periphery (cm)	SR female: 4533 ± 213.6<br>NR female: 4443 ± 219.9<br>Male: 4369 ± 190	($F(2,52)=0.1557$, $P>0.05$) ns
Total time in periphery (sec)	SR female: 842.9 ± 8.129<br>NR female: 831.9 ± 10.46<br>Male: 838.2 ± 7.93	($F(2,52)=0.3882$, $P>0.05$) ns
Number of entries into periphery	SR female: 32.05 ± 4.62<br>NR female: 37 ± 5.75<br>Male: 30.06 ± 3.45	($F(2,52)=0.5692$, $P>0.05$) ns
Number of pokes into periphery	SR female: 5.32 ± 1.13<br>NR female: 4.72 ± 0.72<br>Male: 6.83 ± 1.13	($F(2,52)=1.121$, $P>0.05$) ns
Distance (cm) in center	SR female: 622.1 ± 85.91<br>NR female: 722.4 ± 102.5<br>Male: 615.4 ± 66.42	($F(2,52)=0.4776$, $P>0.05$) ns
Total time (sec) in center	SR female: 57.06 ± 8.13<br>NR female: 68.07 ± 10.46<br>Male: 61.77 ± 7.93	($F(2,52)=0.3882$, $P>0.05$) ns
Number of entries into center	SR female: 32.00 ± 4.61<br>NR female: 37.00 ± 5.78<br>Male: 30.06 ± 3.45	($F(2,52)=0.5707$, $P>0.05$) ns
Number of pokes into center	SR female: 16.79 ± 1.95<br>NR female: 15.72 ± 1.82<br>Male: 24.28 ± 2.54	$F(2,52)=4.771$, $P<0.05$) *
